# Supplementary material for: Role of mTORC1 Signaling in Regulating the Immune Function of Granulocytes in Teleost Fish
Source: Int J Mol Sci. 2023 Sep 6;24(18):13745. doi: 10.3390/ijms241813745 (PMC10530975; doi:10.3390/ijms241813745)
Supplement: Supplementary file 1 [file ijms-24-13745-s001.zip › ijms-2559271-supplementary.pdf]

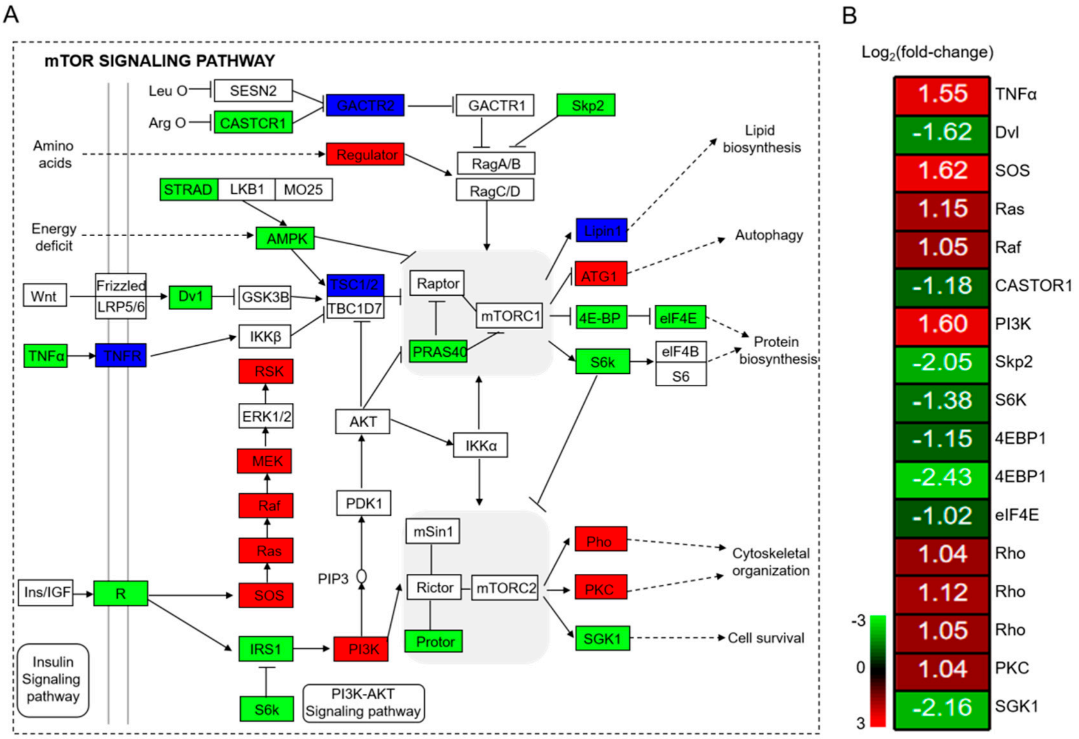

Figure S1. Effect of in vitro treatment of granulocytes with RAPA on mTOR signaling pathways. (**A**) Both of red, green, and deep blue shading boxes represent molecules of the mTOR pathway identified in head kidney granulocytes of largemouth bass. And the red boxes indicate the up-regulated DEGs in this pathway, the green boxes indicate the down-regulated DEGs in this pathway, and the deep blue boxes indicate both up-regulated and down-regulated DEGs in this pathway. (**B**) Differential expression genes involved in the mTOR pathway were analyzed after RAPA treatment. The color gradient represents highly up-regulated (red) to highly down-regulated (green) genes.

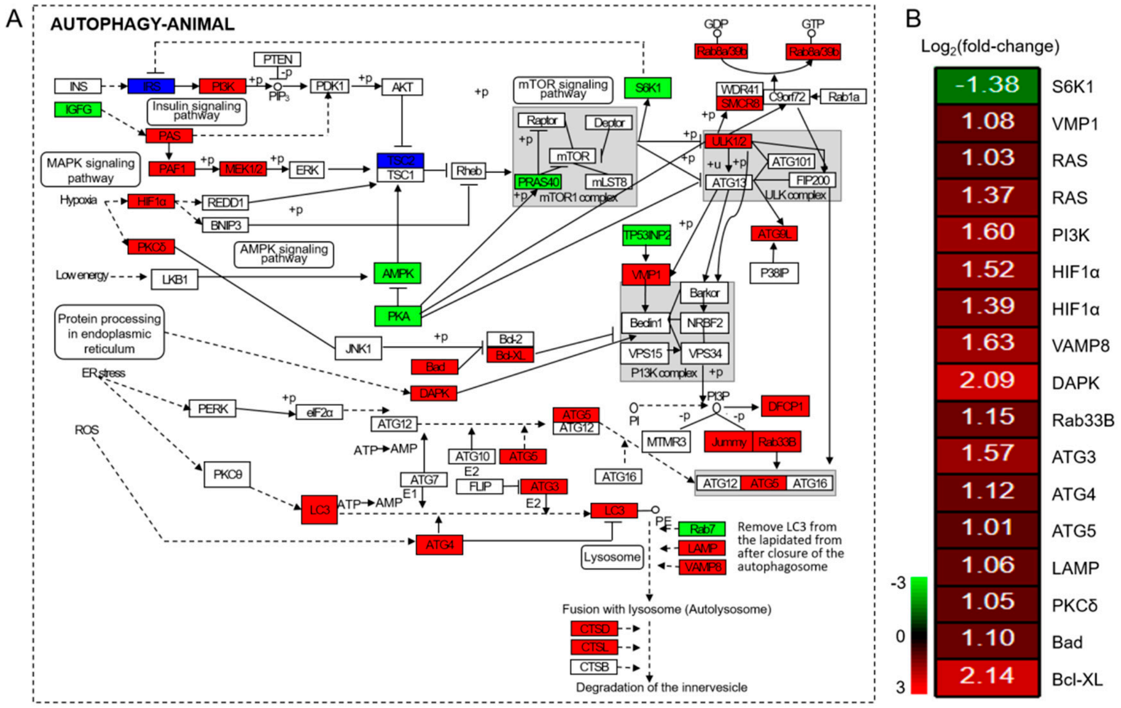

Figure S2. Effect of in vitro treatment of granulocytes with RAPA on autophagy signaling pathways and cell autophagy. (**A**) Both of red, green, and deep blue shading boxes represent molecules of the autophagy pathway identified in head kidney granulocytes of largemouth bass. And the red boxes indicate the up-regulated DEGs in this pathway, the green boxes indicate the down-regulated DEGs in this pathway, and the deep blue boxes indicate both up-regulated and down-regulated DEGs in this pathway. (**B**) Differential expression genes involved in the autophagy pathway were analyzed after RAPA treatment. The color gradient represents highly up-regulated (red) to highly down-regulated (green) genes.

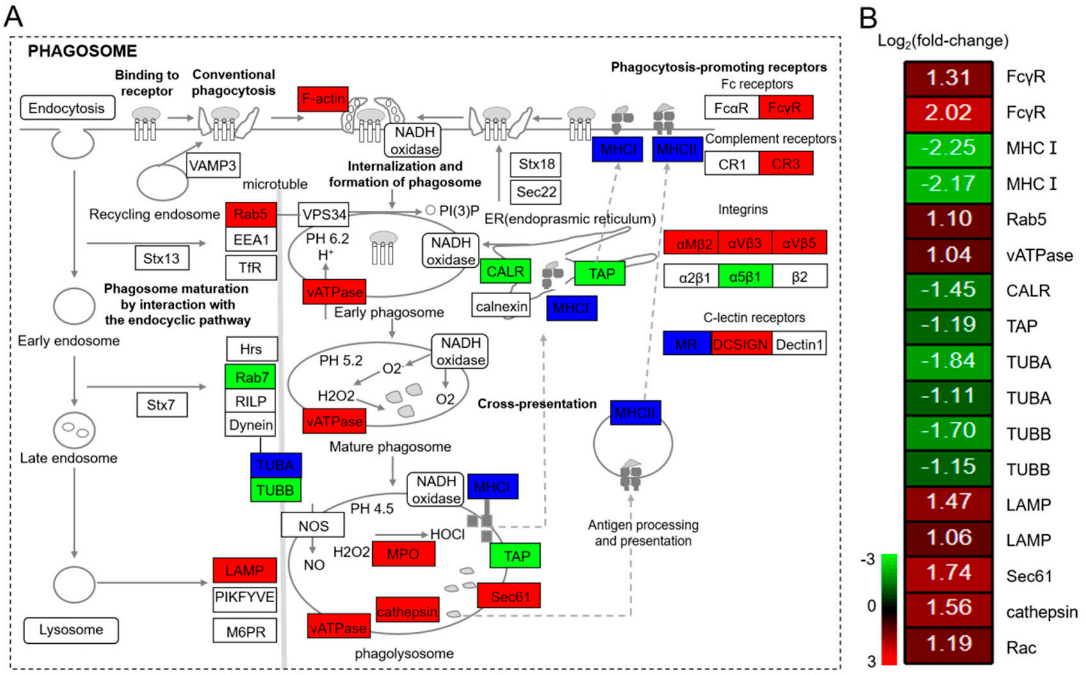

Figure S3. Effect of in vitro treatment of granulocytes with RAPA on phagosome signaling pathways. (**A**) Both of red, green, and deep blue shading boxes represent molecules of the phagosome pathway identified in head kidney granulocytes of largemouth bass. And the red boxes indicate the up-regulated DEGs in this pathway, the green boxes indicate the down-regulated DEGs in this pathway, and the deep blue boxes indicate both up-regulated and down-regulated DEGs in this pathway. (**B**) Differential expression genes involved in the phagosome pathway were analyzed after RAPA treatment. The color gradient represents highly up-regulated (red) to highly down-regulated (green) genes.

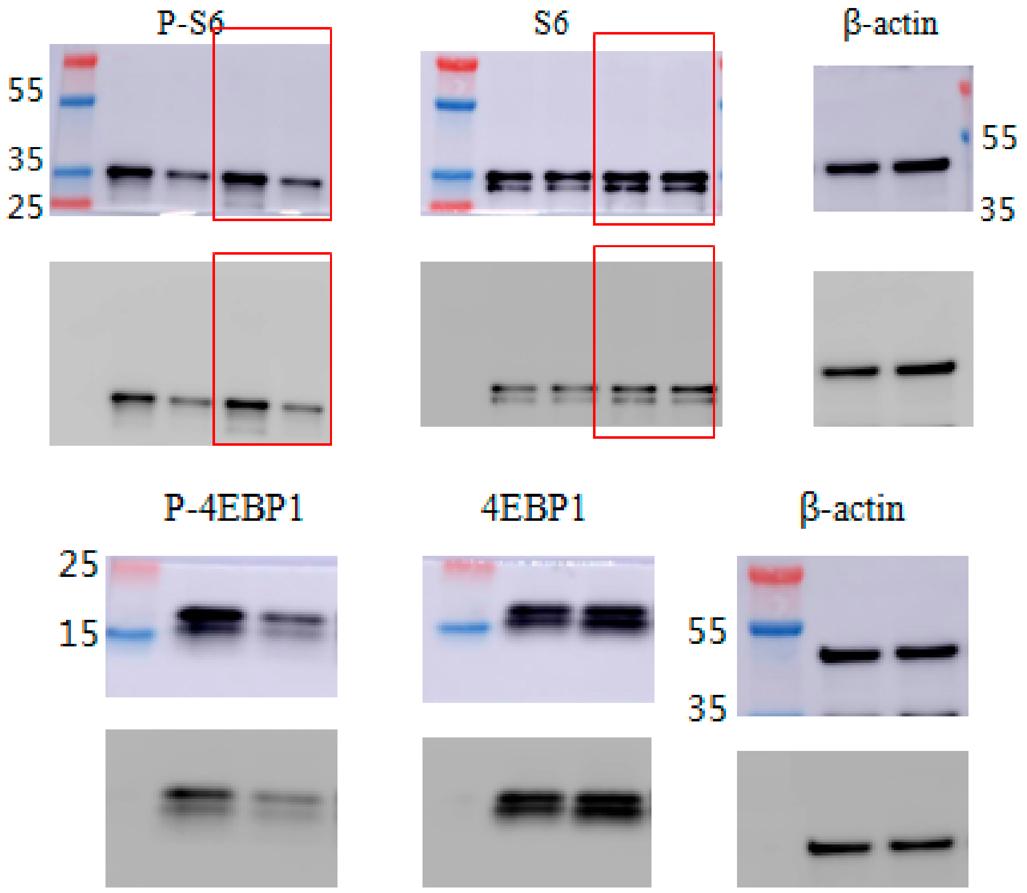

Figure S4. Original images of the western blot analyses in the figure 1E (Upper) and 1F (Lower).
